# Promoter-Autonomous Functioning in a Controlled Environment using Single Molecule FISH

**DOI:** 10.1038/srep09934

**Published:** 2015-05-28

**Authors:** Sami Hocine, Maria Vera, Daniel Zenklusen, Robert H. Singer

**Affiliations:** 1 Department of Anatomy and Structural Biology, Gruss-Lipper Biophotonics Center, Albert Einstein College of Medicine, Bronx, New York, USA; 2 Department of Biochemistry, Université de Montréal, Montreal, Canada

## Abstract

Transcription is a highly regulated biological process, initiated through the assembly of complexes at the promoter that contain both the general transcriptional machinery and promoter-specific factors. Despite the abundance of studies focusing on transcription, certain questions have remained unanswered. It is not clear how the transcriptional profile of a promoter is affected by genomic context. Also, there is no single cell method to directly compare transcriptional profiles independent of gene length and sequence. In this work, we employ a single genetic site for isolating the transcriptional kinetics of yeast promoters. Utilizing single molecule FISH, we directly compare the transcriptional activity of different promoters, considering both synthesis and cell-to-cell variability. With this approach, we provide evidence suggesting promoters autonomously encode their associated transcriptional profiles, independent of genomic locus, gene length and gene sequence.

The ability to regulate and coordinate the transcriptional process is crucial for executing gene expression properly. Much of this regulatory control is exerted over the transcription initiation process, whereby chromatin rearrangements and stepwise-interactions at or near the promoter allow for the assembly of transcriptionally-competent initiation complexes^1^. This process can be broken down into gene-specific and global components, such as the localization of active transcripts to the nuclear periphery^2^,^3^. In yeast, promoter-proximal elements bind gene-specific activators and co-activators that then facilitate the recruitment of the general transcription factors and Pol II^4^. In this way, cells have evolved a streamlined set of basal transcription machinery but maintain the ability to modulate or fine-tune this process on a gene-by-gene basis. This is substantiated by the observation of different transcriptional profiles or “modes” among genes, such as bursting or constitutive behavior^5^,^6^. Bursting expression can be defined as transient periods of gene activity in which multiple rounds of transcription are observed, followed by periods of inactivity. One understanding of this implicates the assembly of highly-stable promoter complexes allowing for successive rounds of initiation. Bursting expression is viewed as a dominant mode of transcription in mammalian systems, but has also been observed for certain genes in yeast^5^,^7^. Alternatively, other genes in both mammalian and yeast systems exhibit what is described as constitutive expression, in which the gene is maintained in an “on” state and short-lived promoter complexes promote single rounds of initiation^5^. In reality, these modes likely represent extremes within a transcriptional spectrum, and a controlled method for quantifying and comparing transcriptional profiles is needed.

Single molecule FISH is an effective and efficient method for measuring both transcriptional activity and steady-state mRNA levels in yeast, and has been used to address a number of biological questions^5^,^8^,^9^,^10^,^11^. While steady-state expression levels and activity at the transcription site become directly accessible by single molecule FISH, it has also been used to infer dynamic properties. Because it is a quantitative technique capable of single cell resolution, the distribution of expression states for a population can be fit to an activation-inactivation computational model^5^. When assessing an individual gene, this approach has yielded information regarding the rate for switching to an “on” state, the rate for switching to an “off” state, and the initiation frequency observed during the “on” state. It is important to note that determination of these values required previous measurements for decay rates from the literature, and a single molecule method for directly comparing transcriptional output among different regulatory regimes provides important data. This approach also fails to consider the contribution of both genomic context and downstream processes to overall expression. Locus, gene length, gene sequence, elongation time, termination time, export and translation rates are all likely to vary depending upon the gene studied. Therefore, a system in which these gene-specific variables are kept constant would allow for a truly direct comparison of transcriptional properties that are conferred solely by the promoter. Here, we use an imaging approach and a controlled system for determining transcriptional activity to characterize promoter-governed transcriptional profiles, keeping all other variables constant.

## Results

### Construction of a reporter for directly comparing transcriptional profiles of yeast promoters

**Figure 1:**
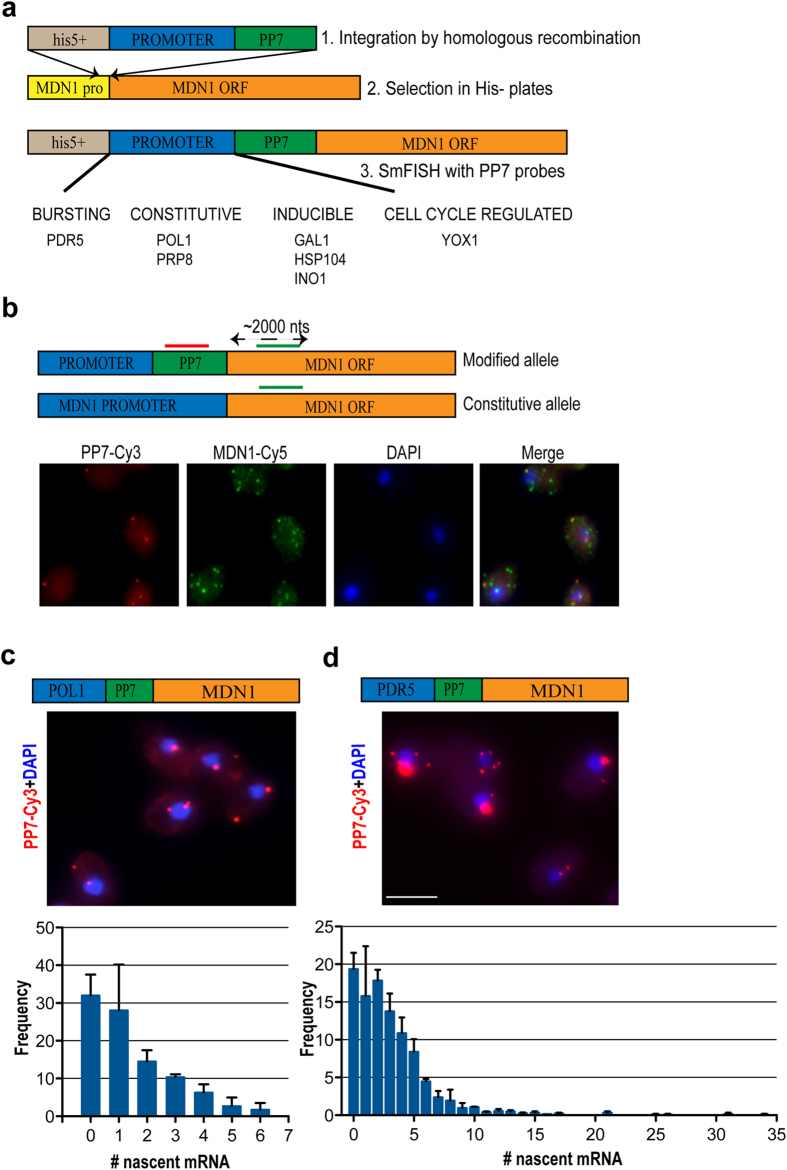
Construction and characterization of a reporter for profiling transcriptional activity. (**a**) Schematic showing the strategy for profiling transcriptional activity for any yeast promoter. Promoter and 24 *PP7* loop sequences are integrated upstream of *MDN1* in diploid yeast, along with a selectable marker. (**b**) Schematic showing the strategy for profiling transcriptional activity from different promoters in the same cell. Red indicates the position of the probe that recognizes the 24 x PP7 loop and green the position of the probe that recognizes the *MDN1* transcripts. PP7V3-Cy3 can be used to visualize only those mRNAs synthesized from the altered allele, as compared to MDN1-Cy3 probes that label mRNAs from both alleles. DAPI signal is used for visualization of the nucleus. (**c-d**) Nascent mRNA distribution for *POL1* and *PDR5* promoters. Schematic showing the modified MDN1 allele. PP7V3-Cy3 used to visualize those mRNAs synthesized from the altered allele. Scale bar is 5 μm. DAPI signal is used for visualization of the nucleus. Plots indicate the nascent mRNA distribution for the altered allele.

We have developed a reporter construct in which different promoters can be shuttled in and out (Fig. 1a). In each case, the promoter drives transcription of *MDN1*, a 14.7 kb essential yeast gene that has been characterized previously in terms of mRNA expression levels^5^. Such a reporter provided several benefits over other single molecule approaches to assess transcriptional profiles. As a result of the length of the *MDN1* transcript, signal detection using FISH probes targeted to the 5’ of the transcript was facilitated, allowing for more accurate quantification of nascent mRNAs than on short genes. Moreover, by keeping the transcribed sequence constant, the reporter represented a normalized method for assessing and comparing the specific contributions of different promoters independent of the variables that are present when comparing endogenous genes to each other.

Promoter sequences were integrated by homologous recombination upstream of one *MDN1* allele in diploid yeast (an essential gene), so as not to affect cell viability. In each case, the promoter was moved from its endogenous location to the *MDN1* locus (Chromosome XII: 349006 – 363738). The insertion of 24 PP7 stem-loops (1200 nts) in the 5’ UTR allowed us to specifically target fluorescent probes to the altered allele and compare it to the endogenous allele to differentiate the promoter-specific effect (Fig. 1b). We have selected various promoters that act through different regulatory mechanisms, including the promoters for the *PDR5*, *PRP8*, *GAL1*, *INO1*, *POL1, HSP104* and *YOX1* genes (Fig. 1a).

### Effects of promoter locus and gene length on transcriptional profiles

Using single molecule FISH, we measured steady-state and nascent mRNA counts and generated representative distributions for a population of cells. We first examined whether this approach was able to recapitulate previously observed transcriptional profiles. As a proof of principle, we compared two promoters known to govern transcription by alternative modes, *POL1* initiating constitutively and *PDR5* initiating in bursts. The *POL1* and *PDR5* promoters show different nascent mRNA distributions indicative of their previously reported modes of transcription^5^. This becomes apparent from the nascent mRNA distributions obtained by transcription site analysis (Fig. 1c, d), and confirms that these promoters function identically when driving *MDN1* expression as compared to their respective endogenous locations. The *POL1* promoter exhibits a nascent transcript distribution ranging from 1 to 6, with a mean of 2.28 nascent mRNA/cell (Fig. 1c and Table 1). The *PDR5* promoter shows a bursting expression, characterized by a wider nascent mRNA distribution, with some transcription sites occupied by as many as 34 transcripts (Fig. 1d and Table 1). For each promoter we calculated the coefficient of variation (cv) as a measure of cell-to-cell variation in transcriptional activity within a population of cells (Table 1). For example, the *PDR5* promoter, when actively transcribing, showed more cell-to-cell variation (cv = 0.88) than *POL1* (cv = 0.57). We then ranked these promoters according to transcriptional output, inferring average initiation intervals by assuming an average Pol II elongation rate of 25 bps/sec, consistent with previous reports in yeast^9^. *PDR5* had a higher transcriptional output in the total population, initiating once every 2.87 minutes, while *POL1* initiated once every 4.96 minutes (Table 1). Therefore, even when driving expression of the *MDN1* reporter at an alternate genomic locus, *POL1* and *PDR5* promoters exhibit the same transcriptional profiles observed in prior studies focusing on the endogenous genes^5^.

For steady-state mRNA levels, initial experiments of the well characterized *PDR5* promoter revealed that, on average, the reporter is expressed at 3.09 mRNAs/cell, as compared to 13.4 mRNAs/cell observed with the endogenous *POL1* gene^5^ (Supplementary Fig. 1a). Such a change in steady-state mRNA expression levels could result from a change in mRNA half-life, induced by the insertion of PP7 stem-loops into the 5’ UTR. The 24xPP7 stem-loop cassette used here contains multiple translation stop codons and is therefore likely to induce nonsense-mediated decay (NMD) when RNAs reach the cytoplasm and are bound by ribosomes. In order to determine whether this was true, we repeated FISH experiments in a strain co-expressing a fluorescent version of the PP7 coat protein. The PP7 coat protein binds the stem loop structures, interferes with translation and likely also inhibits NMD mediated rapid RNA degradation^12^. Consistent with this idea, we observed stabilization of the *POLI* mRNA, up to 8.18/cell when the PP7 coat protein was co-expressed (Supplementary Fig. 1b). Hence decay rates activity was responsible for the decreased mRNA in the cytoplasm. Importantly, the nascent mRNA distribution associated with the *POLI* and *PDR5* promoters recapitulated the transcriptional behavior of the endogenous gene.

### Effects of gene sequence on transcriptional profiles

**Figure 2:**
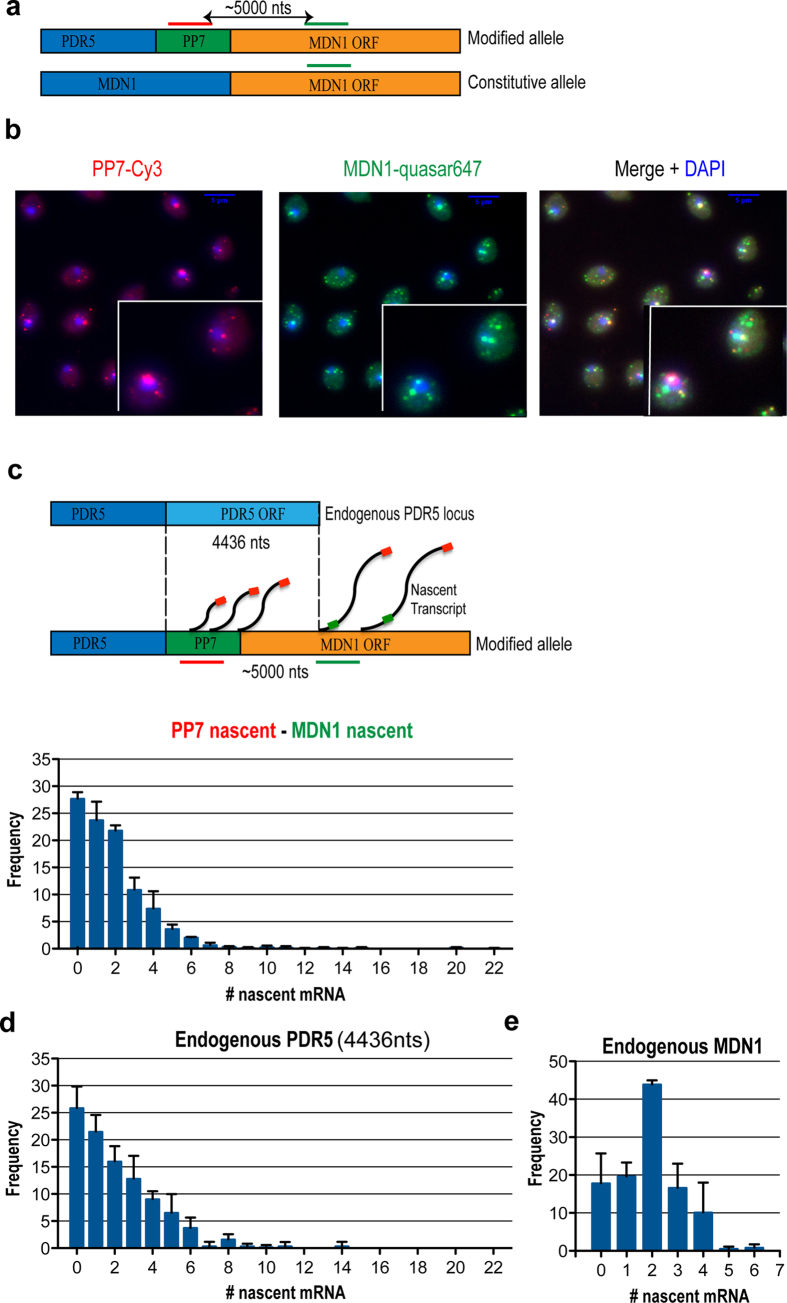
Transciptional profiles depend on the promoter. (**a**) Schematic showing the strategy for profiling transcriptional activity from different promoters in the same cell. Red indicates the position of the probe that recognizes the 24 × PP7 stem-loop and green the position of the probe that recognizes the *MDN1* transcripts. Promoter and 24 × PP7 stem-loop sequences are integrated upstream of *MDN1* in diploid yeast, along with a selectable marker. (**b**) PP7V3-Cy3 is used to quantify only those mRNAs synthesized from the modified allele, as compared to MDN1-quasar 647 probes that label mRNAs from both alleles. DAPI signal is used for visualization of the nucleus. (**c**) Schematic representation of the detection of the red and green signal in the modified allele and correlation with the length of the endogenous PDR5 locus. Nascent mRNA distribution for the modified allele in the first 5000 nts. (**d**) Nascent mRNA distribution for the endogenous *PDR5* gene5. (**e**) Nascent mRNA distribution for the endogenous *MDN1* gene (MDN1-quasar signal with no-signal for PP7).

Having observed that promoters drive certain transcriptional profiles independent of genomic locus and gene length, we next investigated how the sequence of the transcribed region might affect transcriptional output. Comparing the different lengths and sequences of the ectopic transcriptional unit introduces an additional variable that must be considered. In order to determine whether this difference in sequence of the gene itself plays a role in promoter-governed transcriptional profiles, we performed a two-color FISH experiment hybridizing to the 5’ UTR, as well as to a downstream region in the *MDN1* open reading frame that is approximately the same length as the endogenous *PDR5* gene (4,436 nts) (Fig. 2a and b). We determined the number of nascent mRNAs on a region of the *MDN1* transcript that is equivalent to the length of the PDR5 gene. This was accomplished by subtracting the number of nascent transcripts obtained with the downstream fluorescence signal from that of the upstream 5’ UTR signal (PP7) (Fig. 2c), effectively creating a reporter system whereby gene length was the same but sequence was changed. Remarkably, we observed a nearly identical distribution of nascent transcripts when comparing the distribution of the reporter driven off the *PDR5* promoter (Fig. 2c) with that of the endogenous *PDR5* (Fig. 2d)^5^. The frequency of cells not actively transcribing was ~25% and the mRNA distribution was similar between the reporter and endogenous *PDR5*, with the exception of a maximum nascent mRNA count of 22 for the reporter and 14 for endogenous *PDR5*. More importantly, the mean number of nascent mRNA per transcription site was similar (p = 0.9306), with 2.88 for the endogenous *PDR5* sequence and 2.62 for the reporter (Table 1). The cv and the mean initiation interval was also similar for the endogenous *PDR5* and the reporter (Table 1). This result supports the hypothesis that the promoter governs transcriptional profiles independently of the transcribed sequence, and adds further support to the view of promoters as autonomous regulatory units.

### Assessing transcriptional activity of other genes

**Figure 3:**
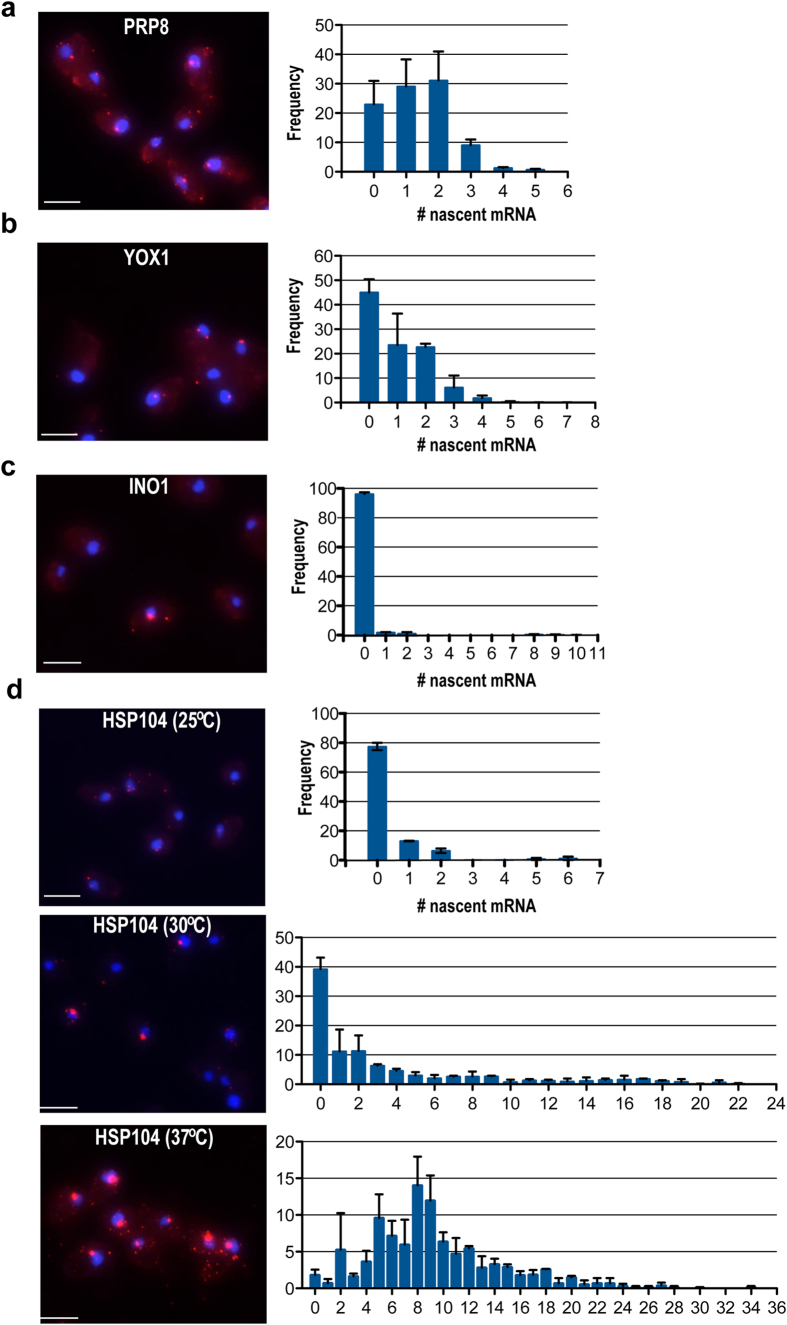
Transcriptional profiles for yeast promoters. (**a-d**) Nascent mRNA distribution for PDR8, YOX1, INO1 and HSP104 promoters. PP7V3-Cy3 used to visualize those mRNAs synthesized from the altered allele. DAPI signal is used for visualization of the nucleus. Scale bar is 5 μm. Plots indicate the nascent mRNA distribution for the altered allele. (**d**) Induction conditions for HSP104 promoter are indicated on the top.

We used the same approach to determine the transcriptional activity of a number of additional yeast promoters, representing a spectrum of different transcriptional control mechanisms (Fig. 1a). We characterized the transcriptional profiles of each promoter by quantifying nascent mRNA distributions for a population of cells (Fig. 3a-d). To assess transcriptional activity while in an active state, we calculated the mean number of nascent transcripts, considering only the values of those cells showing one or more mRNAs, the mean initiation interval and the cv (Table 1). *PRP8* encodes a subunit of the U4/U6-U5 spliceosomal snRNP and is required for the second catalytic step of splicing^13^. The *PRP8* promoter exhibited a constitutive expression with a nascent transcript distribution ranging from 1 to 5, with a mean of 1.79 nascent mRNA/cell and transcription initiating every 6.01 minutes (Fig. 3a). The *YOX1* protein is a transcriptional repressor that binds to early cell cycle boxes (ECBs)^14^,^15^, and only becomes transcriptionally active during the M/G1 transition phase. The active *YOX1* promoter exhibited a nascent transcript distribution ranging from 1 to 7, with a mean of 1.81 nascent mRNA/cell (Fig. 3b). *INO1* encodes the yeast inositol-3-phosphate synthase, and is involved in the synthesis of inositol phosphates and phospholipids^16^. The *INO1* promoter is repressed during growth in low-nitrogen media. Under standard growth conditions, we found that ~95% of the cells did not have any nascent mRNA, hence there was a very small amount of “leaky transcription”. For the subset of cells showing leaky transcription, the *INO1* promoter exhibited bursting expression with a nascent transcript distribution ranging from 1 to 10 per transcription site (Fig. 3c). *HSP104* expression is induced under conditions of cellular stress, such as heat shock. When cells are grown for ten generations at 30 ^o^C, only a subset of cells show active transcription; 40% of cells are transcriptionally silent. Cell transcribing *HSP104* show a high variability in transcriptional activity, consistent with a burst like expression. Calculating the mean number of nascent transcripts showed 5.54 nascent mRNAs per *HSP104* allele and the mean initiation interval was 2.47 minutes (Fig. 3d and Table 1). As expected, a 15-minute shift in the growth temperature from 25 ^o^C to 37 ^o^C increased the mean number of nascent transcripts from 0.30 to 9.49 per cell, leading to a mean initiation interval of 1.16 minutes and a minimum initiation interval of 0.31 minutes (Fig. 3d and Table 1). Finally, consistent with a robust transcriptional response upon temperature shift, we observed higher cell-to-cell variation in expression of the *HSP104* promoter in cells grown at 30 ^o^C (cv = 1.12) compared to cells that underwent the temperature shift to 37 ^o^C (cv = 0.54) (Fig. 3d and Table 1). This further suggests a mixed population of induction states in cells grown at 30 ^o^C.

### Characterization of the transcriptional activity of *GAL1* promoter

**Figure 4:**
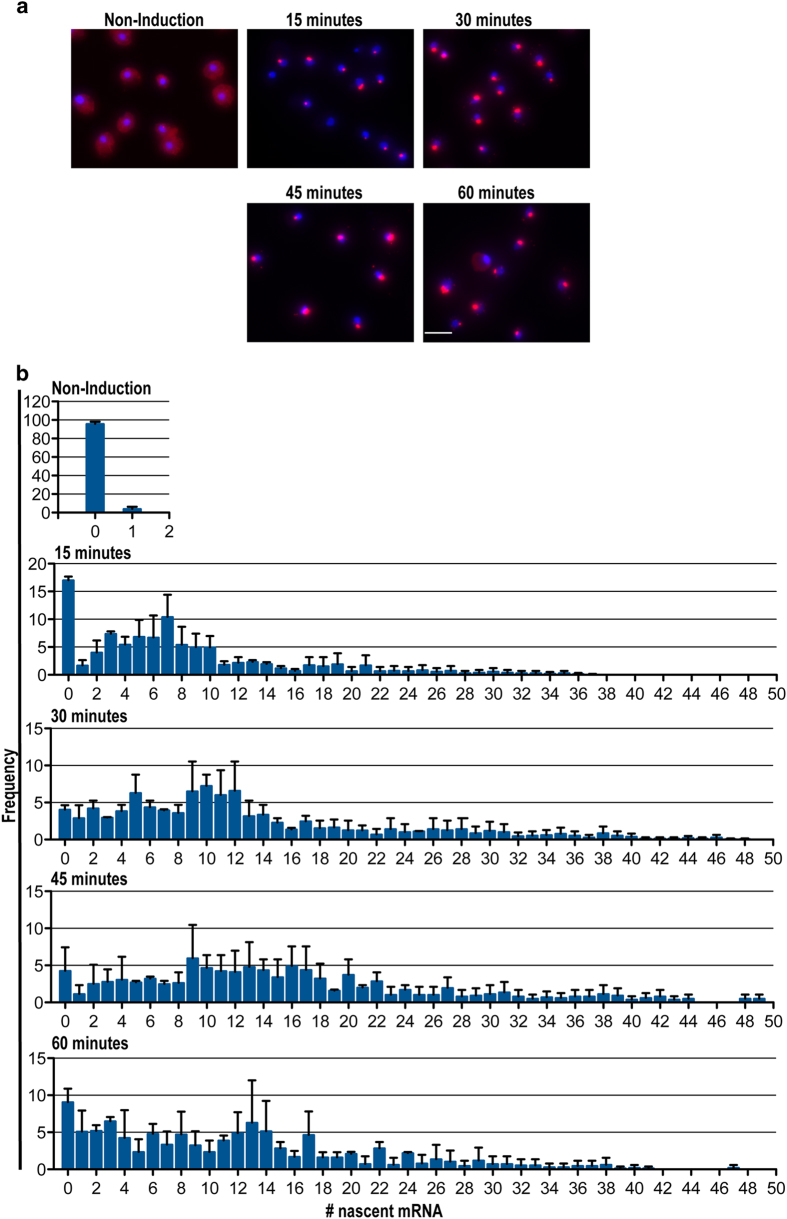
Transcriptional profiles for GAL1 promoter. (**a**) PP7V3-Cy3 used to visualize those mRNAs synthesized from the altered allele. DAPI signal is used for visualization of the nucleus. Scale bar is 5 μm. (**b**) Nascent mRNA distribution for GAL1 promoter at different times of galactose induction.

Next, we used our reporter system to investigate the transcriptional profile of the *GAL1* promoter over time (Fig. 1a). *GAL1* transcription is induced in response to adding galactose to the media^8^. We assessed transcriptional output at 15, 30, 45 and 60 minutes after galactose addition (Fig. 4a,b). In the absence of galactose, only 5% of the cells show active transcription, resulting in a mean number of nascent transcripts of 0.04/cell. By 30 minutes following induction, the percentage of transcribing cells increases to ~95% (Fig. 4b). Similarly, the mean number of nascent mRNA transcripts per cell increases from 9.97 at 15 minutes to 12.98 at 30 minutes. A maximum mean of 15.58 nascent transcripts per cell is reached at 45 minutes, followed by a slight decrease to 13.34 at 60 minutes (Table 1). At peak expression (45 minutes after induction), transcription appears to initiate once every 0.70 minutes on average (Table 1). Initiation however can be much more frequent, as we observe a maximum of 48 nascent RNAs, resulting in an initiation frequency of 0.22 minutes. Calculating the cv at the different time-points of induction also illustrates that there is a large variation in the cell response to the galactose in the media (Table 1). Similarly as for *HSP104*, intrinsic and extrinsic factors are likely to account for the greater cv values observed for these highly regulated promoters. The decrease in transcriptional output, and the increased proportion of cells showing no transcriptional activity at 60 minutes, suggests that the galactose induced transcription response is transient. This observation is similar to induction responses to other stimuli witnessed in higher eukaryotic cells, such estrogen signaling in breast cancer cells^17^. In summary, using the *GAL1* and *HSP104* promoter we demonstrate that this system is suitable to establish a quantitative profile of a transcriptional induction response of any promoter of interest.

## Discussion

Using single molecule FISH, we present a set-up suitable for profiling the transcriptional activity of any yeast gene, where the contribution of the promoter is uncoupled from variables such as genomic locus, gene length, and gene sequence. This system uses diploid *S. cerevisae* cells into which the promoter of one allele of the *MDN1* gene is substituted by any promoter, followed by 24 PP7 stem-loops. We used the PP7 loop sequence to differentiate, using FISH, the modified from the non-modified *MDN1* allele. Although there is a noticeable destabilization of transcripts by the PP7 stem-loops in the cytoplasm, this system is robust to study alternate modes of transcription, as only nascent mRNA counts are considered in the analysis. It is valuable because the same FISH probes and protocol applies for all promoters.

By using the *MDN1* transcription unit as a reporter, we keep the position of the locus, the sequence of the coding region and the gene length constant for each promoter. Therefore, the promoter isolation reporter identifies the transcriptional properties conferred by any yeast promoter. We observed that, in all cases, the promoter-governed synthesis rates recapitulate the behavior of the endogenous gene. Nevertheless, we used the well characterized *PDR5* gene to assess for the influence of the transcribed sequence in the rate of transcription. As shown previously, we demonstrate that the endogenous *PDR5* gene has exhibits bursting transcriptional profile while *MDN1* is transcribed in a constitutive mode. We further showed that the sequence of the *PDR5* promoter is sufficient to induce bursting transcription of the *MDN1* gene, however, with a wider distribution of nascent transcripts than at the endogenous *PDR5*. Since *MDN1* is 3.5 times longer than *PDR5*, we compared it to the number of nascent transcripts expected from an equivalent long gene (the first 5 kb of the *MDN1* gene). In this case, the distribution of the nascent transcripts and the average transcripts per TS were identical for *MDN1* and *PDR5* genes, indicating that the isolated promoter recapitulates the initiation frequency of the endogenous gene. Therefore, the promoter isolation reporter provides a platform to further analyze the contribution of specific sequences to a promoter’s output, and at the same time allows for a direct comparison of different promoter behaviors.

We chose *MDN1* as a reporter because it is the longest gene is *S. cerevisae*, and therefore facilitated signal detection when studying promoters with low transcription frequencies. On a long gene, nascent mRNA will remain at the transcription site for longer periods of time and can be detected using probes binding to the 5’ on the mRNA. Therefore, it provides a better resolution of the initiation profiles for short genes. Furthermore, by keeping variables such as gene length and sequence constant, we are able to directly compare and rank the transcriptional output of different promoters in a controlled way.

An additional benefit of this reporter is that it inherently provides an opportunity to combine imaging approaches with biochemical data, whereby PP7 sequences can be used to isolate target mRNAs in order to assess their mRNP constituents. Thus, the promoter isolation reporter affords a means to purify associated factors that may be responsible for the transcriptional profiles observed using single molecule imaging. This important facet of our work can be appreciated by considering other studies that suggest the fate of several cell cycle and inducible mRNAs in the cytoplasm is determined by the promoter sequence, most probably by factors loaded onto the mRNA at the time of synthesis^11^,^18^,^19^,^20^,^21^,^22^,^23^,^24^.

One of the strengths of single molecule FISH is that it provides quantitative data for single cells. This feature is even more relevant in the case of inducible promoters, like *GAL1* and *HSP104*, as it allows quantitative assessment of all aspects of the induction response, including cell-to-cell variability in response to an external stimulus, resulting in the temporal characterization of promoter activity. For example, the *GAL1* promoter switches from an “off” state to an “on” state in which transcription is initiated more than once per minute on average. Furthermore, by measuring the highest number of nascent mRNAs for each promoter, we can determine the maximum initiation frequency of a given promoter. This system therefore allows determining various aspects of a promoter, including average strength, maximal initiation frequency, variation and timing of transcriptional output in response to different stimuli. For example, in the case of *HSP104*, an interesting next step could be to compare transcriptional profiles observed during heat shock with that of ethanol or sodium arsenate induced stress. Differences in these transcriptional profiles would provide evidence about the different mechanisms involved in each induction response.

The most significant aspect of the work, however, is that we have established a system that controls for many variables long believed to influence gene activity^2^,^3^ including position within the genome, gene length and gene sequence, in additional to certain downstream processes. At least in yeast, the finding that promoters act autonomously provides a new way of thinking about the regulation of gene expression. Future experiments will help to clarify whether promoter autonomy applies globally, or how and why specific subsets of promoters act autonomously while others do not.

## Methods

### Cloning

Promoter sequences were obtained from the Saccharomyces Genome Database (SGD; www.yeastgenome.org). PCR primers were designed to amplify 400–800 bps upstream of the gene. PCR products contained terminal *SacI* and *BamHI* sites, which allowed for the fragment to be cloned into a plasmid containing a his5 selectable marker, an exchangeable promoter region, 24 PP7 repeats, and flanked by regions of MDN1 homology that allow for integration by homologous recombination. Primer sequences are listed in Supplementary Table 1.

### Strain construction

Strains were generated from W303 diploid yeast backgrounds (Supplementary Table 3). 50 mL cultures were grown at 30 °C while shaking at 250 RPM. Cells were grown to an OD_60_f 0.5–1.0, then successively washed in H2O and 1x TE/LiAc. Cells were resuspended in 1mL 1x TE/LiAc, centrifuged, and resuspended a second time in 400 uL 1x TE/LiAc. 50 uL of cells were pelleted and supernatant was removed. 200–400 ng of plasmid DNA was added following digestion with *SpeI/NotI*. 5 μl 10 mg/mL salmon sperm DNA was added, along with 240 uL 50% PEG 3350 (filter sterilized), 36 uL 1.0 M LiAc, and enough water to bring the final reaction volume to 360 uL. Tubes were vortexed and placed in a 42° C water bath for 40 minutes. After heatshock, cells were pelleted, resuspended in 600 uL sterile water, pelleted a second time and finally plated onto synthetic media plates lacking histidine. After two days of growth, single colonies were grown and genomic DNA was harvested using YeaStar Genomic DNA kit (Zymo Research). Colonies were then screened for proper integration of the promoter sequence by PCR.

### Probes for FISH

FISH was performed on all strains using PP7V3 probes (Supplementary Table 2). These 50 nucleotide probes were synthesized using the Applied Biosystems 394 DNA/RNA synthesizer and contained four amino-allyl thymidine that were targets for post-synthesis dye conjugation^25^. Sequences for MDN1 probes for *s.cerevisae* are on Supplementary Table 2.

### FISH coverslip preparation

18 mm circular coverslips (Fisher) were pretreated as follows: Coverslips were first boiled in mass in 500 mL 0.1N HCl (4.13 mL 12.1N HCl added to 500 mL H2O) in a beaker for 10 minutes with period and gentle stirring. Next, coverslips were washed by exchanging acid wash with 200 mL dd H2O and autoclave on standard liquid settings. Coverslips were placed on absorband sterile surfaceand dropped 150 uL of 0.01% poly-L-lysine (Sigma) on each coverslip. After 5 minutes they were washed 3 times with 150 uL dd H2O and air dried.

### Yeast digestion and adhesion

Yeast cultures were grown in appropriate media to OD_600_ of 0.4–0.8. Cells were fixed by adding 8 mL of 32% paraformaldehyde to 42 mLs of culture (final concentration = 4% paraformaldehyde) and shaking at room temperature for 45 minutes. Cells were washed 3 times with 10 mL ice cold 1X buffer B (1.2M sorbitol and 100mM potassium phosphate buffer (pH = 7.5)) and resuspended in 500 μL sphereoblast buffer (1X buffer B containing 20 mM VRC (ribonucleoside-vanadyl complex from NEB) and 25U lyticase enzyme per OD of cells). Digestion of *S. pombe* was performed by adding 0.5 mg zymolyase and 10 mg lysing enzyme from *T. harzianum* to the sphereoblast buffer instead of lyticase and incubated at 30° C for 8 minutes. Next, digested cells were centrifuged at 3500 RPM for 5 minutes, resuspend in 500 uL 1X buffer B by gently pipetting up and down. One hundred and fivety uL of digested cells were added to each lysine-coated coverslip, and incubate at 4° C for 30 minutes and stored in 5 mL 70% EtOH at –20° C.

### Hybridization

Mounted coverslips were rehydrated by adding 2 mL 2X SSC at room temperature for 5 minutes twice. Coverslips were washed with 2 mL hybridization mix, containing 40% formamide/2X SSC at room temperature for 5 minutes. Four ng of labeled probe and 10 μL of 10 mg/μL E. coli tRNA/ssDNA (1:1) mix and dry by speed-vac were added. Twelve uL of solution F (160 uL formamide, 2 uL 1.0 M NaHPO4 (pH = 7.5) and 38 uL dd H2O) were added per coverslip to the dried probe, and heat at 95° C for 3 minutes. A equal volume of solution H (40 μL 20X SSC, 40 μL 10 mg/mL BSA, 20 μL 100 mM VRC and 100 uL dd H2O) was added to the probe mix and vortex. Twenty uL of probe-containing hybridization mix was added per coverslip, and coverslips were placed cell-side facing down onto glass plates coated with parafilm, sealed with an additional layer of parafilm to maintain moisture, cover with aluminum foil and place in a 37° C dry incubator for 3 to 12 hours. After two washes with 2 mL hybridization mix at 37° C for 15 minutes, one with 0.1% Triton X-100 in 2X SSC at room temperature for 15 minutesand two additional washes with 1X SSC for 15 minutes at room temperature, nucleus was stained with2 mL 1X PBS/0.5 μg/mL DAPI for 2 minutes at room temperature. A final wash with 2 mL 1X PBS for 5 minutes at room temperature was done before mounting on standard microscope slides by adding a small drop (~15 μL) of ProLong Gold antifade reagent (Invitrogen) and inverted coverslips, cell-side facing down.

### Double FISH

Because of the different annealing properties of 20-mer and 50-mer smFISH probes, two color smFISH with PP7-V3 cy3 probes and *MDN1*-Q670 (Biosearch technologies, see probe list) was done in two steps. The first step with PP7-V3 probes is the same as described in hybridization (see above). After the second wash with hybridization mix, covers were incubated for 30 minutes with 10% formamide/2X SSC at room temperature. The same procedure was repeated for *MDN1* probes with a final concentration of formamide of 10% instead of 40%.

### Imaging

Images were acquired using an Olympus BX61 widefield epi-fluorescence microscope outfitted with a 100X/1.35 NA UPlanApo objective. Samples were visualized using the Chroma 41007 filter (Cy3), Chroma 31000 filter (DAPI), and DIC. Metamorph (Molecular Devices) was used as the acquisition software in combination with a CoolSNAP HQ CCD camera (Photometrics). Z-sections were acquired at 200 nm intervals over an optical range of 4.0 um. Exposure times for each z-section include 1200 ms (Cy3), 200 ms (Cy5) and 25 ms (DAPI). Cell volumes are then assembled by maximum intensity projections of z-sections using Metamorph. Localize, developed by Dr. Daniel Larson, was used to detect, locate and quantify intensities for fluorescent spots based on a two-dimensional Gaussian mask algorithm detailed previously^5^,^26^. Cell-segmentation software was used to identify cell and nuclear boundaries, and nascent mRNA counts were determined by dividing the transcription site intensity by the mean single transcript intensities, and rounding up or down to the nearest whole number^5^. The mean initiation interval = ((lenth of the gene in Kb/mean # of nascent mRNA)/1.5 Kb per minute)). The maximum initiation interval = ((lenth of the gene in Kb/maximun # of nascent mRNA)/1.5 Kb per minute)).

## Additional Information

**How to cite this article**: Hocine, S. *et al.* Promoter-Autonomous Functioning in a Controlled Environment using Single Molecule FISH. *Sci. Rep.* **5**, 9934; doi: 10.1038/srep09934 (2015).

## Supplementary Material

Supplementary Information

## Figures and Tables

**Table 1:** The mean number of nascent mRNAs is determined for each distribution. (*)To calculate mean initiation intervals assuming an elongation rate of 25 bps/sec9. cv = coefficient of variation. (-) Small fraction of cells with active transcript. (n/a) not applicable. Times of mean initiation interval and minimum initiation interval are indicated in minutes.

**YEAST STRAIN**	**total N**	**% cell “on” state**	man # of nascent(^*^)	**man initiation interval**	**minimum initiation interval**	**cv**
POLI	912	80.4	2.28	4.96	1.52	0.57
PDR5	815	80.7	3.79	2.87	0.31	0.88
MND1-PP7 (PDR5)	995	78.7	2.62	1.21	n/a	0.84
Endogenous PDR5		76.3	2.88	1.04	n/a	0.76
PRP8	651	76.2	1.79	6.01	1.52	0.55
YOX1	912	54.7	1.81	6.13	1.52	0.51
INO1	640	3.8	0.12	90.00	n/a	-
HS25	407	20.7	0.30	37.27	0.46	-
HS30	609	60.8	5.54	2.47	0.31	1.12
HS 37 (15 min)	739	98.2	9.49	1.16	n/a	0.54
GAL no-induction	432	4.1	0.04	381.63	n/a	-
GAL 15 min	670	82.0	9.97	1.35	0.29	0.74
GAL 30 min	725	94.4	12.98	0.88	0.23	0.65
GAL 45 min	603	94.7	15.58	0.70	0.22	0.60
GAL 60 min	690	90.9	13.34	0.82	0.22	0.66
