# Supplementary material for: Promoter-Autonomous Functioning in a Controlled Environment using Single Molecule FISH
Source: Sci Rep. 2015 May 28;5:9934. doi: 10.1038/srep09934 (PMC4446897; doi:10.1038/srep09934)
Supplement: Supplementary Information [file srep09934-s1.pdf]

Supplementary Information

Promoter-Autonomous Functioning in a Controlled Environment using Single Molecule FISH

Sami Hocine[1*], Maria Vera[1*], Daniel Zenklusen[1,2] & Robert H. Singer[1]

Supplementary Figure

Supplement Figure-1 Singer

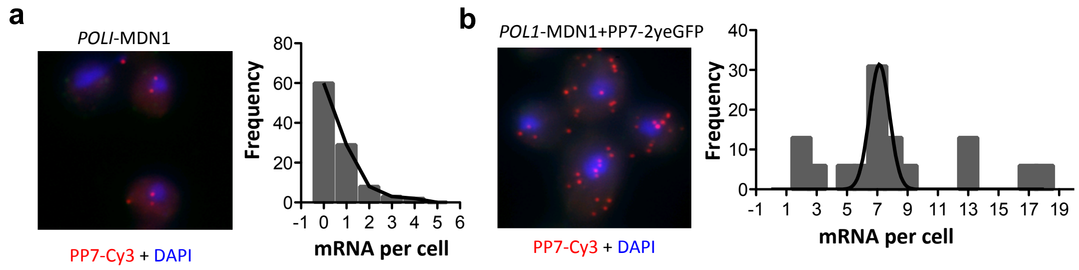

Supplementary Fig. 1: Steady-state mRNAs are destabilized by the presence of 24 PP7.

(a-b) PP7V3-Cy3 FISH used to visualize only those mRNAs synthesized from the altered *POL1* allele. DAPI signal is used for visualization of the nucleus. Distribution of cytoplasmic mRNA counts for the *POL1* promoter in the absence (a) and presence (b) of the PP7 coat protein fused to 2yeGFP.

Supplementary Tables.

Supplementary Table 1: Oligos

Oligo Name	Oligo Sequence
1003 PDR5 SacI fwd	ATATAT GAGCTC GCAGCACCTCGTTGGCGCAGT
1004 PDR5 BamHI rev	ATATAT GGATCC AGTCTTTCGAACGAGCGGATACG
797 POL1 pro SacI fwd	ATATATGAGCTCTACATGAACTGACCGAAATTGC
798 POL1 pro BamHI rev	ATATATGGATCCTTTCCACTGTTTATTTTATGCCTTGATACT
799 YOX1 pro SacI fwd	ATATATGAGCTCAAACCTTCTACTGACTCCCCT
800 YOX1 pro BamHI rev	ATATATGGATCCTCAACCACGGAAAGCGTTCGGA
762 INO1 pro SacI fwd	ATA TAT GAG CTC CGA TGT GCC CTT GAT GGA CAA C
763 INO1 pro BamHI rev	ATA TAT GGA TCC TAG GAA CCC GAC AAC AGA ACA AGC
764 PRP8 pro SacI fwd	ATA TAT GAG CTC CAA TAT ATT ACC GGT TAG CGC AAC
765 PRP8 pro BamHI rev	ATA TAT GGA TCC TCT TTC TTT GAC GCT ATT TT
729 HSP104 pro SacI fwd	ATATATGAGCTC TCGTGGTAGTGTTTGCTGTTCC
733 HSP104 pro BamHI rev	ATATATGGATCCATATTCTGTATATTTTATGGTACGTGTAGTTG
726 GAL1 pro SacI fwd	ATATATGAGCTC TCAGTAATACGCTTAACTGCTCATTGC
734 GAL1 pro BamHI rev	ATATATtGGATCCTATAGTTTTTTCTCCTTGACGTTAAAG

SupplementaryTable 2: Probes

50 mer probes	**Sequence**
PP7V3	G**T**A CCT TAG GA**T** CTA ATG AAC CCG GGA A**T**A CTG CAG CCA GCG AGC CCA **T**A
SPMDN1 702	**T**AC AGC TTG TG**T** CAA CAC ACC AGG **T**TG CCA CTC GAA T**T**C TCC AGG TTT **T**G
SPMDN1 1477	**T**CT CAC CAA C**T**A GAA GCA ATG G**T**T CGT TTG TTT GAA CAC C**T**G CAG CCA GC**T** G
SPMDN1 1984	C**T**A GTA ATA CCC AGT G**T**C CGG AAC GAA CCG CC**T** TCA CCA AAG CAC CC**T** CA
SPMDN1 2395	T**T**C GGC TCA ATG **T**TC GAA CGG TA**T** AGT GTG GTC T**T**T GAC CAG CTC CA**T** CA
SPMDN1 1624	GCA A**T**G GAA GAC CAA G**T**G TGG AAG CG**T** TGA TAG GTT TA**T** AAC CGC CAA GCA **T**A

***bold nucleotides denote dye conjugation sites**

20 mer probes for MDN1 (5000 nts)Probe (5'-> 3')
1 actctttcaagtctacatct
2 cagttgaacatcttcggcta
3 aaactatccctcagcttttg
4 ccgtcactccattcaaataa
5 gtcctcatagcttgaatcag
6 gcgatatttcatcaagtaga
7 tcaagaacggaatcatctgc
8 ctggctccaaaacactattt
9 tctgctaacaacaaactcct
10 aaggctatcggatgaaccct
11 gaaagttttctgaagccgtt
12 gggttcatggttgcgaagaa
13 cttttttaccgtaatctcca
14 ctatttcttaacgctggaga
15 atggtacccatatttcagta
16 acatcgttgaaatcttccat
17 ccttgaggatacgatcatgt
18 agcaagatctttcaagtcct
19 tcttgccaaaccattcagag
20 aatgacaccacttgttgcat
21 ttcaacccatgcaagtatgt
22 acttgttctgtattttcgga
23 gcaccttgaattaaggcagt
24 gggcgtcaataaaaaccatt
25 taagcagtgttatttgtacc
26 atcattttcgttttcagcta
27 acattctgttctaagtgact
28 ttccaaatcgtcaccacata
29 cgttcgtttcaatttgttgt

30 gtaattcatcctgagtcaca
31 ggtattttgaacattccgac
32 atgaagactgagcatctgga
33 gtgggggcggttaagttaaa
34 ccttactaagtttgaggcag
35 tggcttgtgtacttgcatag
36 cctggactaccttcaagtaa
37 gcggtgattaaacttgtttt
38 tatttcctgtaatattggca

Supplementary Table 3: Yeast strains

ySH001	PDR5-his5-PP7-MDN1	W303 diploid
ySH002	INO1-his5-PP7-MDN1	W303 diploid
ySH003	ASH1-his5-PP7-MDN1	W303 diploid
ySH004	PRP8-his5-PP7-MDN1	W303 diploid
ySH005	YOX1-his5-PP7-MDN1	W303 diploid
ySH006	POL1-his5-PP7-MDN1	W303 diploid
ySH007	GAL1-his5-PP7-MDN1	W303 diploid
ySH020	HSP104-his5-PP7-MDN1	W303 diploid
